# Chemical Characterization of Outdoor and Subway Fine (PM_2.5–1.0_) and Coarse (PM_10–2.5_) Particulate Matter in Seoul (Korea) by Computer-Controlled Scanning Electron Microscopy (CCSEM)

**DOI:** 10.3390/ijerph120202090

**Published:** 2015-02-13

**Authors:** Sang-Hoon Byeon, Robert Willis, Thomas M. Peters

**Affiliations:** 1 Department of Environmental Health, College of Health Sciences, Korea University, Seoul 136-703, Korea; E-Mail: shbyeon@korea.ac.kr; 2 National Exposure Research Laboratory, U.S. Environmental Protection Agency, Research Triangle Park, NC 27711, USA; E-Mail: willis.robert@epa.gov; 3 Department of Occupational and Environmental Health, University of Iowa, 100 Oakdale Campus, Iowa City, Iowa 52242, USA

**Keywords:** subway, CCSEM, particulate matter, passive sampling, indoor

## Abstract

Outdoor and indoor (subway) samples were collected by passive sampling in urban Seoul (Korea) and analyzed with computer-controlled scanning electron microscopy coupled with energy dispersive x-ray spectroscopy (CCSEM-EDX). Soil/road dust particles accounted for 42%–60% (by weight) of fine particulate matter larger than 1 µm (PM_2.5–1.0_) in outdoor samples and 18% of PM_2.5–1.0_ in subway samples. Iron-containing particles accounted for only 3%–6% in outdoor samples but 69% in subway samples. Qualitatively similar results were found for coarse particulate matter (PM_10–2.5_) with soil/road dust particles dominating outdoor samples (66%–83%) and iron-containing particles contributing most to subway PM_10–2.5_ (44%). As expected, soil/road dust particles comprised a greater mass fraction of PM_10–2.5_ than PM_2.5–1.0_. Also as expected, the mass fraction of iron-containing particles was substantially less in PM_10–2.5_ than in PM_2.5–1.0_. Results of this study are consistent with known emission sources in the area and with previous studies, which showed high concentrations of iron-containing particles in the subway compared to outdoor sites. Thus, passive sampling with CCSEM-EDX offers an inexpensive means to assess PM_2.5–1.0_ and PM_10-2.5_ simultaneously and by composition at multiple locations.

## 1. Introduction

Airborne particulate matter (PM) has been associated with adverse respiratory and cardiovascular health effects [1,2,3]. Understanding the chemical and physical properties of fine and coarse ambient particles is essential to the understanding of emission sources, transport, and deposition mechanisms as well as the health impact of particles deposited in the respiratory system [4].

Numerous studies have focused on the biological effects of transition metals (iron, vanadium, nickel, chromium, copper and zinc) in PM because of their ability to generate reactive oxygen species (ROS) which can cause oxidative stress, cell death, biological aging and diseases [5,6,7,8,9,10,11,12,13,14,15,16,17,18]. Iron (Fe) is of particular interest in this study because of the high concentration of Fe-rich particles in subways and because iron is usually present at much higher concentrations in ambient air than other transition metals [19]. The role of iron in mediating health effects from exposure to PM has been explored in a number of studies. Donaldson *et al.* [20] and Valavanadis [21] demonstrated that iron released from airborne PM can stimulate the generation of hydroxyl radicals by Fenton-type reactions, causing extensive oxidative damage to cellular macromolecules. Strong associations between iron and oxidative stress and pulmonary inflammation were also reported [22,23,24,25]. ROS activity in rat alveolar macrophages exposed *in vitro* to Denver (CO, U.S.A.) PM correlated highest (among nine distinct source types) with an unidentified iron source in the Denver airshed [22,23,24,25,26]. Shafer *et al.* [27] concluded that transition metals, particularly iron, are major factors mediating the ROS-activity of water extracts of PM collected in Lahore (Pakistan). In contrast to the above studies, Lay *et al.* [28] studied the effects of inhaled iron oxide particles on alveolar epithelial permeability in healthy human subjects and found no appreciable alteration of alveolar epithelial permeability, lung diffusing capacity, or pulmonary function.

The U.S. Environmental Protection Agency (EPA) currently regulates airborne concentrations of PM_10_ and PM_2.5_ (particulate matter with aerodynamic diameter <10 µm and <2.5 µm, respectively). In 2006, the EPA proposed to replace the PM_10_ standard with PM_10–2.5_, defined as the difference between PM_10_ and PM_2.5_ (“coarse” particles). This change would have eliminated the duplication in regulation of particles <2.5 µm in both PM_10_ and PM_2.5_ and may have prevented adverse health effects associated with exposure specifically to ambient coarse particles. Although the EPA ultimately retained the PM_10_ standard, there remains a need to understand the sources and composition of coarse PM and its potential health effects [29,30].

Traditionally, air quality studies have employed filter-based sampling and bulk analytical methods to determine ambient PM concentrations and average aerosol composition. However, computer-controlled scanning electron microscopy coupled with energy dispersive x-ray spectroscopy (CCSEM-EDX) can provide information on the size, morphology, and elemental composition of individual particles, which can enable source identification. This technique was developed in the late 1970s and early 1980s and has been applied in numerous studies to characterize aerosols in the environment and for source apportionment [31,32,33,34,35,36,37]. Mamane *et al.* [4] investigated how many particles are needed to statistically represent a sample and concluded that the major particle class abundances and average class compositions converged to within a few percent from final values after analyzing several hundred particles. Kang *et al.* [38] and Jung *et al.* [39] employed a variation of CCSEM-EDX called low-Z particle electron probe X-ray microanalysis (low-Z particle EPMA) to characterize aerosol composition in the Seoul (Korea) subway system.

Passive sampling is an inexpensive way to measure particulate matter in many locations simultaneously and thus offers a way to improve exposure assessment [40,41,42,43]. Wagner and Leith, 2001 developed the UNC passive aerosol sampler in which ambient PM concentrations are estimated from: (1) the surface loading of particles collected onto a substrate over time, and (2) knowledge of the flux, or rate of transfer, of these particles to the sampler. In the Wagner-Leith method, surface loading is determined by microscopy, by counting and sizing particles that have deposited on the substrate, and flux is estimated from a semi-empirical model that is a function of the aerodynamic diameter of a particle, da. Passive sampling combined with CCSEM-EDX has been used in recent studies in the U.S. to characterize particles in urban air sheds [44,45,46].

Concerns about the public health impact of subway aerosols [47,48,49,50,51,52,53,54], have motivated a number of air quality studies in subway systems around the world [39,47,49,52,55,56,57]. Two of these studies [38,39] were conducted in the Seoul subway system: Kang *et al.* [38] monitored particles in the Hyehwa subway station which are the most frequently encountered with relative abundances in the range of 61%–79%., while Jung *et al.* [39] characterized Fe-containing particles in four subway stations (Jegi, Chungmuro, Yangjae, and Seouldae) which decrease as the distance of sampling locations from the tunnel increases. Samples collected at the platform in subway stations with platform screen doors (PSDs) that limit airmixing between the platform and the tunnel showed marked decreases in relative abundances of Fe-containing particles, clearly indicating that Fe-containing subway particles are generated in the tunnel [58]. Jung *et al.* [39] collected particles with a three-stage PM_10_ sampler, obtaining particles in two size fractions: 10 µm to 2.5 µm; and 2.5 µm to 1 µm. Kang *et al.* [38] used a 7-stage May cascade impactor with particles collected onto Ag foil. Disadvantages of these collection methods are that an air pump is needed to “actively” pull air through the sampler and particles may bounce from collection substrates and pass erroneously to subsequent stages if substrates are not treated properly.

The goal of our study was to use passive sampling in conjunction with CCSEM-EDX to compare the chemical compositions of fine and coarse particle matter in outdoor settings to that in the Jegi subway station in Seoul, South Korea. Passive sampling requires substantially less effort than active sampling. Thus, this technique may allow substantially more samples to be collected than “active” sampling for similar or lower cost.

## 2. Experimental Section 

**Figure 1:**
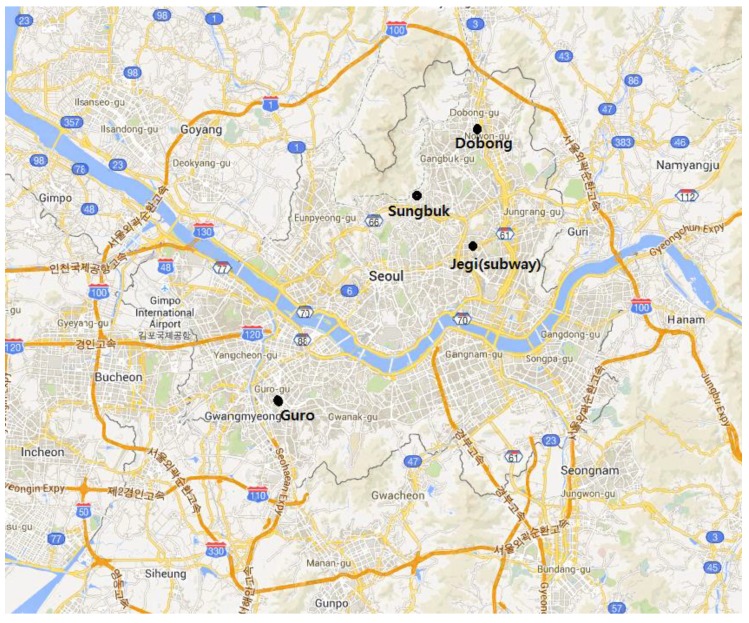
Passive sampling locations in Seoul, Korea.

Passive samplers were deployed over seven-day periods in June 2010 in four locations (Dobong, Sungbuk, Guro and Jegi subway station) in Seoul, South Korea (Figure 1). Dobong, Sungbuk and Guro are residential sites located along a line running northeast to southwest through the center of Seoul. Jegi is a subway station on Line 1 of Seoul’s multiline subway system. These sites were selected to provide samples with contrasting chemical make-up. Each site has its own characteristics. The Sungbuk site was located near the traffic road, the Dobong site was located near the forest and incineration and the Guro site was located beside building construction. Ten samples were collected, with two samples per location, including two blanks. The Jegi samples were collected from the subway’s platform which does not employ platform screen doors to limit mixing between the subway tunnel and the platform [39]. The passive samplers used in this work were identical to those described by Ault *et al.* [44]. Briefly, particles were collected on polycarbonate substrates, which provide a flat, featureless substrate for CCSEM analysis. The substrates were mounted on an SEM stub below a protective mesh cap as described by Wagner and Leith [43]. The entire assembly was placed within a protective shelter [59].

Collected particles were analyzed with the Personal SEM™ (PSEM) (Aspex Corporation, Delmont, PA, USA) and with a Mira3 Field Emission SEM (Tescan USA Inc., Cranberry Township, PA, USA). Both instruments are equipped with secondary and backscattered electron detectors and thin-window, energy-dispersive X-ray detectors (EDX) that enable X-ray detection of carbon and heavier elements. Both instruments use proprietary software to perform CCSEM analysis of the sample and to enable off-line review and processing of CCSEM data. More details on particle analysis by CCSEM are provided in Mamane *et al.* [4].

For the present study, the PSEM software was configured to analyze particles between 1 and 18 μm (physical diameter) and to retain only those particles with aerodynamic diameter da between 1 and 10 µm. The da of each particle is estimated from the particle’s physical diameter and EDX composition. The following SEM parameters were used: 20 kV accelerating voltage, magnification of 720×, 16 mm working distance, backscatter detection mode, and an EDX acquisition time of 2.5 sec, sufficient to acquire a robust X-ray spectrum. CCSEM was conducted in the point analysis mode, whereby the electron beam was focused at the center of each particle while X-rays were acquired. The analysis time per sample was about 2–3 h in which ~80% of the exposed collection area was analyzed and samples before SEM analysis were coated by carbon coater. A total of 12,429 particles in the eight field samples were analyzed by CCSEM and particle diameters were also calculated by PSEM software. The particles in each sample were classified using rules based on their elemental composition into 19 chemical classes or particle types shown in Table 1 (the class labeled “Others” contains several minor classes). Results of the CCSEM analysis were broken down by particle size into PM_2.5–1.0_ (“fine” particles between 1 and 2.5 µm, aerodynamic diameter) and PM_10–2.5_ (“coarse” particles between 2.5 and 10 µm). Following Wagner and Leith [43], a volume shape factor (1.6) was used to convert the projected area diameter from SEM imaging to an equivalent volume diameter. This volume diameter was then converted to aerodynamic diameter assuming spherical shape and applying a particle density estimated from analysis of x-ray spectrum assuming that the particle was in the form of an oxide [44,60].

**Table 1:** Average relative abundances and standard deviations (weight %) of PM_2.5–1.0_ composition by particle types from the outdoor and subway sites. Two samples were collected in each location. Values in italics indicate the abundance of individual particle types that were grouped into broader categories.

Particle Type		Weight % (Standard Deviation)			
		Dobong	Sungbuk	Guro	Jegi (subway)
Soil/road dust	Sum of	42 (± 23)	49 (± 5)	60 (± 2)	18 (± 7)
	*Al-Si, Al-Si-K, Al-Si-Mg*	*24*	*31*	*36*	*8*
	*Ca/S, Ca/Si, Ca-Mg, Ca-rich*	*14*	*13*	*18*	*9*
	*Si-rich*	*4*	*6*	*6*	*1*
Iron-containing		3 (± 0.4)	4 (± 0.1)	6 (± 0.1)	69 (± 8)
Carbonaceous		26 (± 5)	21 (± 1)	24 (± 2)	8 (± 1)
Aluminum		3 (± 2)	2 (± 0.1)	2 (± 1)	2 (± 1)
Secondary nitrate/sulfates	Sum of	21 (± 27)	18 (± 8)	4 (± 1)	0.3 (± 0.3)
	*Na/Cl, Na/S, Na-rich*	*6*	*11*	*2*	*0*
	*S-rich*	*15*	*7*	*2*	*0*
Others		4 (± 0.2)	5 (± 2)	4 (± 1)	3 (± 1)

## 3. Results and Discussion

**Figure 2:**
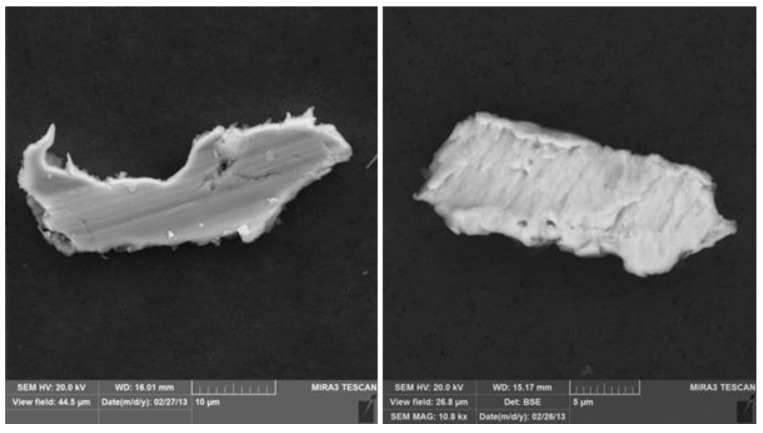
Stainless steel particles collected in Jegi subway station showing surface features characteristic of wear processes. Such particles were typically larger than 10 µm and were unique to the Jegi site. The EDX spectra showed Fe, Cr, and Ni.

**Figure 3:**
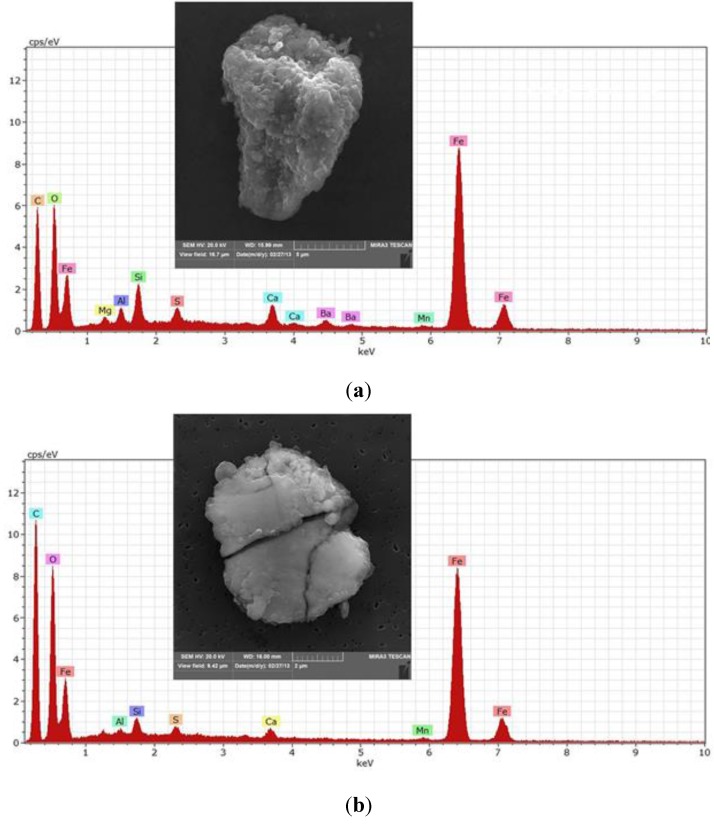
(**a**) Fe-Ca-Si particles from the Jegi subway station. (**b**) Fe-Ca-Si particles were found in much higher concentration in the subway site compared to the outdoor sites. These particles may originate in friction between the brake block and the train wheels. The spectrum in Figure 3a also shows sulfur, barium and manganese.

Results are summarized in Table 1 and Figure 2 for fine particles (PM_2.5–1.0_) and in Table 2 and Figure 3 for coarse particles (PM_10–2.5_). Following Jung *et al.* [39], the chemical composition of the PM_2.5–1.0_ samples was sorted into six major particle-type categories: soil/road dust, iron-containing, carbonaceous, aluminum, secondary nitrates/sulfates, and other. Soil/road dust included Al-Si, Al-Si-K, Al-Si-Mg, Ca/S, Ca/Si, Ca-Mg, Ca-rich, and Si-rich components. Particles in the iron-containing class had EDX spectra in which iron x-rays comprised at least 20% of the EDX spectrum. Carbon x-rays comprised at least 50% of the EDX spectrum for the carbonaceous class. Aluminum particles were distinguished from soil/road dust particles by their absence of silicon. Secondary nitrates/sulfates included Na/Cl, Na-rich and S-rich components.

As shown in Table 1, the order of abundance of these major particle types for the outdoor samples was: soil/road dust > carbonaceous > secondary nitrates/sulfates > others > iron-containing > aluminum. Soil/road dust particles were the greatest contributor to fine particle mass at the outdoor locations, accounting for 42% (Dobong) to 60% (Guro) of PM_2.5–1.0_. Within the soil/road dust classification, aluminum silicate particles accounted for the greatest percentage by weight followed by calcium-containing particles and finally silicon-rich particles for both outdoor and subway samples. Carbonaceous particles accounted for 21%–26%, secondary nitrates/sulfates were 4%–21%, others were 4%–5%, iron-containing were 3.5%–5.8% and aluminum particles were 2%–3%.

Some inter-site differences among the outdoor PM_2.5–1_ samples are notable (Table 1). Guro aerosol had more soil/road dust by weight (60%) and less sodium/sulfur-rich mass (4%) compared to Dobong and Sungbuk. We attribute the similar composition of PM_2.5-1_ aerosol samples in Sungbuk and Dobong to the relative proximity of these two sites (7.8 km), although several mountains are located around Dobong. Guro, however, is located south of the Han River at a distance of 16.7 km from Sungbuk. Many buildings were under construction within 1.0km of the Guro site during sampling which may account for the higher concentration of soil/road dust. The highest concentration of S-rich particles (presumably sulfate) was observed in Dobong (15%). The emission source for these particles is unknown but may originate from a garbage incineration plant located about 2.5 km from the sampling location.

**Table 2:** Average relative abundances and standard deviations (weight %) of PM_10–2.5_ composition by particle types from the outdoor and subway sites. Two samples were collected in each location. Values in italics indicate the abundance of individual particle types that were grouped into broader categories.

Particle Type		Weight % (Standard Deviation)			
		Dobong	Sungbuk	Guro	Jegi (subway)
Soil/road dust	Sum of	66 (± 1)	83 (± 2)	83 (± 4)	36 (± 2)
	*Al-Si, Al-Si-K, Al-Si-Mg*	*51*	*59*	*66*	*23*
	*Ca/S, Ca/Si, Ca-Mg, Ca-rich*	*12*	*18*	*10*	*12*
	*Si-rich*	*4*	*5*	*6*	*1*
Iron-containing		5 (± 3)	4 (± 0.2)	3 (± 1)	44 (± 4)
Carbonaceous		16 (± 4)	6 (± 1)	7 (± 2)	8 (± 5)
Aluminum		3 (± 2)	2 (± 1)	3 (± 2)	5 (± 7)
Secondary nitrates/sulfates	Sum of	3 (± 1)	2 (± 0.3)	1 (± 1)	2 (± 1)
	*Na/Cl, Na/S, Na-rich*	*1*	*1*	*1*	*1*
	*S-rich*	*2*	*1*	*0*	*1*
Others		7 (± 5)	4 (± 1)	4 (± 2)	6 (± 1)

The Na/Cl, Na/S and Na-rich content of PM_2.5–1.0_ was substantially higher at Sungbuk compared to that observed at other sites. These particles may originate from road salt applied in the winter near the site. Particles collected in the Jegi subway station were strikingly different from those collected at the outdoor sites. The order of abundance of the major particle types for the Jegi subway samples was: iron-containing > soil/road dust > carbonaceous > others > aluminum > secondary nitrates/sulfates. These samples were dominated by iron-containing particles (69% of PM_2.5–1.0_ by weight compared to 3%–6% at the outdoor sites, a factor of 12 to 23 times higher).

These results are consistent with previous subway air quality studies in which iron-containing particles comprised the most abundant particle class by weight [39,47,49,52,55,56,57]. Seaton *et al.* [49] reported that iron oxide particles comprised 67% by weight of PM_2.5_ in samples collected in London Underground stations. In the Jegi subway station, Jung *et al.* [39] found that 70.9% (±8.0) of iron-containing particles by number were in the size range of 2.5 µm to 1 µm, which is in close agreement with our finding of 66% (±9).

Iron-containing particles in subways are generated mainly from mechanical wear and friction processes at rail-wheel-brake interfaces, and at the interface between catenaries providing electricity to subway trains and pantographs attached to trains [39]. Jung *et al.* characterized aerosol composition at different locations within Seoul subway stations to convincingly show that iron-containing subway particles are generated in the subway tunnels.

As shown in Table 2, the order of abundance of the major particle types for the three outdoor sites was soil/road dust > carbonaceous > others > iron-containing > aluminum > secondary nitrates/sulfates. As expected, soil/road dust particles were the greatest contributor to coarse particle mass in the outdoor samples, accounting for 66% to 83% of PM_10–2.5_. Within the soil/road dust classification, aluminum silicate particles accounted for the greatest percentage by weight followed by calcium-containing particles and silicon-rich particles. Carbonaceous particles comprised 6%–16% by mass, others were 4%–7%, iron-containing were 3%–5%, aluminum particles accounted for 2%–3%, and secondary nitrates/sulfates were 1%–3% by mass.

In outdoor samples, soil/road dust particles accounted for a higher proportion of mass for PM_10–2.5_ than for PM_2.5–1.0_. In contrast, secondary nitrates/sulfates and carbonaceous particles accounted for a higher proportion of mass for PM_2.5–1.0_ than for PM_10–2.5_. These observations are consistent with coarse mode particles being derived primarily from crustal material and fine mode having large portions of nitrates/sulfates and carbonaceous particles from other sources.

In contrast to fine particles, where abundances were most similar between the closest sites (Dobong and Sungbuk), abundances of coarse particles were most similar at the most distant sites (Sungbuk and Guro), while the Dobong samples had a higher weight percent of coarse carbonaceous particles. Compared to fine particles, coarse particles can have short atmospheric lifespans [61]; thus, differences among sites are not surprising. SEM-EDX analysis of the outdoor samples revealed more than a three-fold increase in the weight percent of pollens and plant debris particles (a subset of the carbonaceous class) in the Dobong samples relative to Sungbuk and Guro.

Subway samples showed a different order of abundance: iron-containing > soil/road dust > carbonaceous > others > aluminum > secondary nitrates/sulfates. Within the soil/road dust class in subway samples, particles containing aluminum silicates accounted for the greatest percentage by weight followed by calcium-rich particles and finally silicon-rich particles. Iron-containing and soil/road dust particles dominated coarse mass in the subway samples (44% and 36%, respectively).

In subway samples, iron-containing particles were the greatest contributor to mass for PM_2.5–1.0_ (69%) and for PM_10–2.5_ (44%). The smaller size fraction of particles showed a higher concentration of iron-containing particles. Also in the subway samples, the mass fraction of coarse soil/road dust particles abundance was twice the fine mode mass fraction.

Kang *et al.* [62] reported that urban PM_10_ samples collected far away from subway stations contained 4.6% by number of iron-containing particles, which agrees with our number concentration of 4%, averaged over Sungbuk, Dobong, and Guro samples.

The most striking difference in PM composition between sites is the abundance of iron-containing particles in the Jegi subway site relative to the outdoor locations (Table 1 and Table 2). Manual and computer-controlled SEM-EDX were used to investigate differences in iron particle composition and/or morphology between sites. The majority of Fe-rich particles were iron oxides as evidenced by the presence of oxygen in the EDX spectra (not shown).

Compared to the outdoor sites, samples collected in the Jegi subway had higher concentrations of stainless steel particles as well as particles rich in iron, calcium and silicon (Fe-Ca-Si class). Stainless steel particles from the subway station were typically larger than 10 µm and many showed surface features consistent with wear (Figure 2). The source of these particles is unknown but may be attributable to wear from the subway cars.

The abundance of Fe-Ca-Si particles in subway samples, also noted by Jung *et al.* [39], is attributed by Sitzmann *et al.* [55] to friction between the train wheels and the brake blocks (composed of iron, glass fibers and CaCO3). Figure 3a and 3b show images and spectra of subway particles representing the Fe-Ca-Si class. The particle in Figure 3a is decorated on its surface with many fine iron-rich particles smaller than a few hundred nanometers. Kang *et al.* [38] hypothesized that nanosized particles on the surface of large Fe-containing particles are formed by the condensation of gaseous iron species from the sparking between the third rail and the electricity guide of subway trains, in a process similar to that of arc welding particles. And nonosized particles can also be formed by mechanical processes of sliding and wear at the brake-wheel and wheel-rail interfaces.

**Figure 4:**
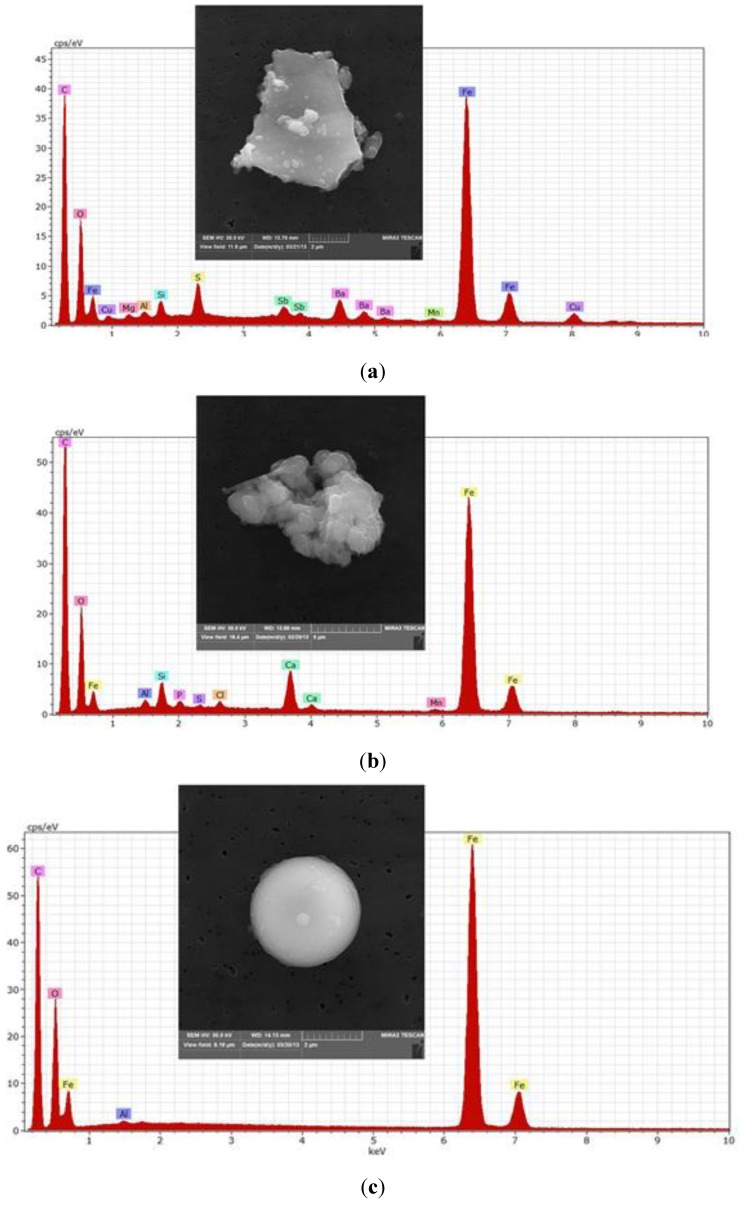
(**a**) Likely automotive brake wear particle from Dobong with Ba, S, Sb, and Cu. (**b**) Fe-Ca-Si particle from Dobong, similar to those found in subway. (**c**) Iron-oxide sphere from Guro, typically associated with steel production.

Electron micrographs and spectra of iron-containing particles found at the outdoor sites are provided in Figure 4. The particle shown in Figure 4a (Dobong) was identified as automotive brake wear from Dobong. That shown in Figure 4b is a Fe-Ca-Si particle from Dobong of unknown origin, similar in composition to those identified in the subway. Lastly, the particle shown in Figure 4c is an iron oxide sphere from Guro, presumed to originate from steel processing operations in the vicinity.

## 4. Conclusions 

This study demonstrates the ability of low-cost passive sampling coupled with CCSEM-EDX analysis to identify differences in chemical composition of fine and coarse PM across multiple sites within an urban area. The chemical compositions of particles collected outdoors at three sites in Seoul, South Korea were similar. For PM_2.5–1.0_ the similarities were greatest for the sites that were closer to each other, Dobong and Sungbuk. However, for PM_10-2.5_, Sungbuk and Guro were most similar in composition. Soil/road dust particles dominated both PM_10–2.5_ and PM_2.5–1.0_ in outdoor samples, accounting for a larger fraction of PM_10–2.5_ mass than PM_2.5–1.0_ mass. The Jegi subway station aerosol was quite different in composition from the outdoor samples. Iron-containing particles accounted for only a small fraction of outdoor mass (3%–6%) but represented a large fraction (44%–69%) of mass in subway aerosol samples. Iron-containing particles accounted for a larger fraction of PM_2.5–1.0_ than PM_10–2.5_. Our results from the Jegi station agree with previous studies, which have reported enhanced concentrations of iron-containing particles in subway stations.

## References

[1] Chen Y., Yang Q., Krewski D., Shi Y., Burnett R.T., McGrail K. (2004). Influence of relatively low level of particulate air pollution on hospitalization for COPD in elderly people. Inhal. Toxicol..

[2] Fung K.Y., Khan S., Krewski D., Chen Y. (2006). Association between air pollution and multiple respiratory hospitalizations among the elderly in Vancouver, Canada. Inhal. Toxicol..

[3] Krewski D., Rainham D. (2007). Ambient air pollution and population health: Overview. J. Toxicol. Environ. Health Pt. A.

[4] Mamane Y., Willis R., Conner T. (2001). Evaluation of computer-controlled scanning electron microscopy applied to an ambient urban aerosol sample. Aerosol. Sci. Tech..

[5] Carter J.D., Ghio A.J., Samet J.M., Devlin R.B. (1997). Cytokine production by human airway epithelial cells after exposure to an air pollution particle is metal-dependent. Toxicol. Appl. Pharmacol..

[6] Costa D.L., Dreher K.L. (1997). Bioavailable transition metals in particulate matter mediate cardiopulmonary injury in healthy and compromised animal models. Environ. Health Perspect..

[7] Goldsmith A., Ning H.D.Y., Carroll-Ann W.G.A.I. (1998). Analysis of air pollution particulate-mediated oxidant stress in alveolar macrophages. J. Toxicol. Environ. Health Pt. A.

[8] Kodavanti U.P., Hauser R., Christiani D.C., Meng Z.H., McGee J., Ledbetter A., Richards J., Costa D.L. (1998). Pulmonary responses to oil fly ash particles in the rat differ by virtue of their specific soluble metals. Toxicol. Sci..

[9] Ghio A.J.D., Devlin R.B. (2001). Inflammatory lung injury after bronchial instillation of air pollution particles. Amer. J. Respir. Crit. Care Med..

[10] Li N., Sioutas C., Cho A., Schmitz D., Misra C., Sempf J., Wang M., Oberley T., Froines J., Nel A. (2003). Ultrafine particulate pollutants induce oxidative stress and mitochondrial damage. Environ. Health Perspect..

[11] Hetland R.B., Cassee F.R., Refsnes M., Schwarze P.E., Lag M., Boere A.J.F., Dybing E. (2004). Release of inflammatory cytokines, cell toxicity and apoptosis in epithelial lung cells after exposure to ambient air particles of different size fractions. Toxicol. Vitro.

[12] Valavanidis A., Fiotakis K., Bakeas E., Vlahogianni T. (2005). Electron paramagnetic resonance study of the generation of reactive oxygen species catalysed by transition metals and quinoid redox cycling by inhalable ambient particulate matter. Redox Rep..

[13] Valavanidis A., Fiotakis K., Vlachogianni T. (2008). Airborne particulate matter and human health: Toxicological assessment and importance of size and composition of particles for oxidative damage and carcinogenic mechanisms. J. Environ. Sci. Health C-Carcin..

[14] Chen L.C., Lippmann M. (2009). Effects of metals within ambient air particulate matter (PM) on human health. Inhal. Toxicol..

[15] Gualtieri M., Mantecca P., Corvaja V., Longhin E., Perrone M.G., Bolzacchini E., Camatini M. (2009). Winter fine particulate matter from Milan induces morphological and functional alterations in human pulmonary epithelial cells (A549). Toxicol. Lett..

[16] Akhtar U.S., McWhinney R.D., Rastogi N., Abbatt J.P., Evans G.J., Scott J.A. (2010). Cytotoxic and proinflammatory effects of ambient and source-related particulate matter (PM) in relation to the production of reactive oxygen species (ROS) and cytokine adsorption by particles. Inhal. Toxicol..

[17] Wei H., Wei D., Yi S., Zhang F., Ding W. (2011). Oxidative stress induced by urban fine particles in cultured EA.hy926 cells. Hum. Exp. Toxicol..

[18] Ghio A.J., Carraway M.S., Madden M.C. (2012). Composition of air pollution particles and oxidative stress in cells, tissues, and living systems. J. Toxicol. Environ. Health Pt. B.

[19] Lu S., Liu D., Zhang W., Liu P., Fei Y., Gu Y., Wu M., Yu S., Yonemochi S., Wang X. (2015). Physico-chemical characterization of PM_2.5_ in the microenvironment of Shanghai subway. Atmos. Res..

[20] Donaldson K., Brown D.M., Mitchell C., Dineva M., Beswick P.H., Gilmour P., MacNee W. (1997). Free radical activity of PM_10_: Iron-mediated generation of hydroxyl radicals. Environ. Health Perspect..

[21] Valavanidis A. (2000). Generation of hydroxyl radicals by urban suspended particulate air matter. The role of iron ions. Atmos. Environ..

[22] Smith K.R., Veranth J.M., Hu A.A., Lighty J.S., Aust A.E. (2000). Interleukin-8 levels in human lung epithelial cells are increased in response to coal fly ash and vary with the bioavailability of Iron, as a function of particle size and source of coal. Chem. Res. Toxicol..

[23] Quinlan G.J., Evans T.W., Gutteridge J.M.C. (2002). Iron and the redox status of the lungs. Free Radical Biol. Med..

[24] Zelikoff J.T., Schermerhorn K.R., Fang K., Cohen M.D., Schlesinger R.B. (2002). A role for associated transition metals in the immunotoxicity of inhaled ambient particulate matter. Environ. Health Perspect..

[25] Zhang Y., Schauer J.J., Shafer M.M., Hannigan M.P., Dutton S.J. (2008). Source apportionment of *in vitro* reactive oxygen species bioassay activity from atmospheric particulate matter. Environ. Sci. Technol..

[26] Zhang Y.M., Napier-Munn T.J. (1995). Effects of particle size distribution, surface area and chemical composition on Portland cement strength. Powder Technol..

[27] Shafer M.M., Perkins D.A., Antkiewicz D.S., Stone E.A., Quraishi T.A., Schauer J.J. (2010). Reactive oxygen species activity and chemical speciation of size-fractionated atmospheric particulate matter from Lahore, Pakistan: An important role for transition metals. J. Environ. Monit..

[28] Lay J.C., Zeman K.L., Ghio A.J., Bennett W.D., Lay J.C., Zeman K.L., Ghio A.J., Bennett W.D. (2001). Effects of inhaled iron oxide particles on alveolar epithelial permeability in normal subjects. Inhal. Toxicol..

[29] Brunekreef B., Forsberg B. (2005). Epidemiological evidence of effects of coarse airborne particles on health. Eur. Resp. J..

[30] Chang H.H., Peng R.D., Dominici F. (2011). Estimating the acute health effects of coarse particulate matter accounting for exposure measurement error. Biostatistics.

[31] Kim D., Hopke P.K. (1988). Source apportionment of the El Paso aerosol by particle class balance analysis. Aerosol Sci. Tech..

[32] Dzubay T.G., Mamane Y. (1989). Use of electron microscopy data in receptor models for PM_10_. Atmos. Environ..

[33] Vander Wood T.B., Brown R.S. (1992). The application of automated scanning electron microscopy/energy dispersive X-ray spectrometry to the identification of sources of lead-rich particles in soil and Dust. Environmental Choices Technical Supplement.

[34] Johnson D.H., Hunt A., Beard M., Allen Iske S. (1995). Analysis of lead in urban soils by computer assisted SEM/EDX—Method development and early results, in lead in paint, soil and dust: Health risks, exposure studies, control measures, measurement methods, and quality assurance. Special Technical Publication.

[35] Katrinak K.A., Anderson J.R., Buseck P.R. (1995). Individual particle types in the aerosol of Phoenix, Arizona. Environ. Sci. Technol..

[36] Jambers W., van Grieken R. (1997). Single particle characterization of inorganic suspension in Lake Baikal, Siberia. Environ. Sci. Technol..

[37] Conner T.L., Norris G.A., Landis M.S., Williams R.W. (2001). Individual particle analysis of indoor, outdoor, and community samples from the 1998 Baltimore particulate matter study. Atmo. Environ..

[38] Kang S., Hwang H., Park Y., Kim H., Ro C.U. (2008). Chemical compositions of subway particles in Seoul, Korea determined by a quantitative single particle analysis. Environ. Sci. Technol..

[39] Jung H.-J., Kim B., Ryu J., Maskey S., Kim J.-C., Sohn J., Ro C.-U. (2010). Source identification of particulate matter collected at underground subway stations in Seoul, Korea using quantitative single-particle analysis. Atmos. Environ..

[40] Noll K.E., Fang K.Y.P., Watkins L.A. (1988). Characterization of the deposition of particles from the atmosphere to a flat plate. Atmos. Environ..

[41] Brown R.C., Wake D., Thorpe A., Hemingway M.A., Roff M.W. (1994). Preliminary assessment of a device for passive sampling of airborne particulate. Ann. Occup. Hyg..

[42] Vinzents P.S. (1996). A passive personal dust monitor. Ann. Occup. Hyg..

[43] Wagner J., Leith D. (2001). Passive aerosol sampler. Part I: Principle of operation. Aerosol Sci. Tech..

[44] Ault A.P., Peters T.M., Sawvel E.J., Casuccio G.S., Willis R.D., Norris G.A., Grassian V.H. (2012). Single-particle SEM-EDX analysis of iron-containing coarse particulate matter in an urban environment: Sources and distribution of iron within Cleveland, Ohio. Environ. Sci. Technol..

[45] Kumar P., Hopke P.K., Raja S., Casuccio G., Lersch T.L., West R.R. (2012). Characterization and heterogeneity of coarse particles across an urban area. Atmos. Environ..

[46] Mukerjee S., Willis R.D., Walker J.T., Hammond D., Norris G.A., Smith L.A., Welch D.P., Peters T.M. (2012). Seasonal effects in land use regression models for nitrogen dioxide, coarse particulate matter, and gaseous ammonia in Cleveland, Ohio. Atmos. Pollut. Res..

[47] Chillrud S.N., Epstein D., Ross J.M., Sax S.N., Pederson D., Spengler J.D., Kinney P.L. (2004). Elevated airborne exposures of teenagers to manganese, chromium, and iron from steel dust and New York City’s Subway System. Environ. Sci. Technol..

[48] Karlsson H.L., Nilsson L., Möller L. (2005). Subway particles are more genotoxic than street particles and induce oxidative stress in cultured human lung cells. Chem. Res. Toxicol..

[49] Seaton A., Cherrie J., Dennekamp M., Donaldson K., Hurley J.F., Tran C.L. (2005). The London underground: Dust and hazards to health. Occup. Environ. Medicine.

[50] Karlsson H.L., Ljungman A.G., Lindbom J., Möller L. (2006). Comparison of genotoxic and inflammatory effects of particles generated by wood combustion, a road simulator and collected from street and subway. Toxicol. Lett..

[51] Bachoual R., Boczkowski J., Goven D., Amara N., Tabet L., On D., Leçon-Malas V., Aubier M., Lanone S. (2007). Biological effects of particles from the Paris subway system. Chem. Res. Toxicol..

[52] Salma I., Weidinger T., Maenhaut W. (2007). Time-resolved mass concentration, composition and sources of aerosol particles in a metropolitan underground railway station. Atmos. Environ..

[53] Gustavsson P., Bigert C., Pollán M. (2008). Incidence of lung cancer among subway drivers in Stockholm. Amer. J. Ind. Med..

[54] Karlsson H.L., Holgersson Å., Möller L. (2008). Mechanisms related to the genotoxicity of particles in the subway and from other sources. Chem. Res. Toxicol..

[55] Sitzmann B., Kendall M., Watt J., Williams I. (1999). Characterisation of airborne particles in London by computer-controlled scanning electron microscopy. Sci. Total Environ..

[56] Furuya K. (2001). Seasonal variation and their characterization of suspended particulate matter in the air of subway stations. J. Trace. Microprobe. Tech..

[57] Aarnio P., Yli-Tuomi T., Kousa A., Mäkelä T., Hirsikko A., Hämeri K., Räisänen M., Hillamo R., Koskentalo T., Jantunen M. (2005). The concentrations and composition of and exposure to fine particles (PM_2.5_) in the Helsinki subway system. Atmos. Environ..

[58] Moreno T., Martins V., Querol X., Jones T., BéruBé K., Minguillón M.C., Amato F., Capdevila M., de Miguel E., Centelles S. (2015). A new look at inhalable metalliferous airborne particles on rail subway platforms. Sci. Total Environ..

[59] Ott D.K., Kumar N., Peters T.M. (2008). Passive sampling to capture spatial variability in PM_10–2.5_. Atmos. Environ..

[60] Sawvel E.J., Willis R., West R.R., Casuccio G.S., Norris G., Kumar N., Hammond D., Peters T.M. (2015). Passive sampling to capture the spatial variability of coarse particles by composition in Cleveland, OH. Atmos. Environ..

[61] Ott D.K., Cyrs W., Peters T.M. (2008). Passive measurement of coarse particulate matter, PM_10–2.5_. J. Aerosol. Sci..

[62] Kang S., Hwang H., Park Y., Kim H., Ro C.U. (2009). Quantitative ED-EPMA combined with morphological information for the characterization of individual aerosol particles collected in Incheon, Korea. Atmos. Environ..

